# Angular‐Adaptive Reconfigurable Spin‐Locked Metasurface Retroreflector

**DOI:** 10.1002/advs.202100885

**Published:** 2021-09-05

**Authors:** Weixu Yang, Ke Chen, Yilin Zheng, Wenbo Zhao, Qi Hu, Kai Qu, Tian Jiang, Junming Zhao, Yijun Feng

**Affiliations:** ^1^ Department of Electronic Engineering School of Electronic Science and Engineering Nanjing University Nanjing 210093 China

**Keywords:** angular‐adaptive retroreflector, omnidirectional space, reconfigurable metasurface, spin‐locked operation

## Abstract

Metasurface retroreflectors, which scatter the incident electromagnetic wave back to incoming direction, have received significant attention due to their compelling advantages of low profile and light weight compared with conventional bulky retroreflection devices. However, the current metasurface retroreflectors still have limitations in wide‐angle and omnidirectional operations. This work proposes a high‐efficiency, wide‐angle, reconfigurable, and omnidirectional retroreflector composed of spin‐locked phase gradient metasurface with a thickness of only 5.2 mm or 0.07 operating wavelength. The reflection phase of constituent meta‐atoms can be controlled dynamically and continuously by altering their orientation states through individually addressing each mechanically rotational meta‐atom, whereas the reflection handedness is kept the same as incidence. Therefore, adaptive and arbitrary momentum can be imparted to the incident wave, providing high‐efficiency retroreflection over a wide continuous range from −47° to 47°. Moreover, such high‐performance retroreflection is extended to omnidirectional level, enabling great degrees of freedom that are unavailable by previous researches. As a proof of concept, a retroreflective metasurface is fabricated and experimentally demonstrated at microwave frequencies. The proposed thin thickness, high efficiency, and reconfigurable metasurface retroreflector can be extended to other frequencies that may offer an untapped platform toward reconfigurable spin‐based retroreflection devices for electromagnetic signal processing.

## Graphical abstract

An angular‐adaptive spin‐locked retroreflector is realized using a reconfigurable metasurface composed of mechanically rotational meta‐atoms. The reflection phase of meta‐atoms can be controlled dynamically by altering the orientation states based on the Pancharatnam‐Berry phase. Therefore, adaptive and arbitrary momentum can be imparted to the incident wave, enabling high‐efficiency retroreflections over a large continuous range in an omnidirectional half‐space.

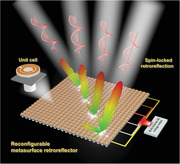

## 1 Introduction

Manipulating the electromagnetic (EM) wave at will has been a long‐held dream since the discovery of Maxwell's equation. Recently, metasurfaces, spatially varying engineered subwavelength‐sized structures, have been used to control the wavefront of EM wave with unprecedented abilities by introducing phase discontinuities across the interface,^[^ ^1^, ^2^, ^3^, ^4^, ^5^ ^]^ leading to various fascinating phenomena and novel planar device components, such as metalenses,^[^ ^6^, ^7^, ^8^ ^]^ beam shapers,^[^ ^9^, ^10^ ^]^ wave plates,^[^ ^11^, ^12^ ^]^ holographic imagers,^[^ ^13^, ^14^ ^]^ and optical encryption.^[^ ^15^ ^]^ The metasurfaces provide a flexible platform to achieve versatile wave functionalities with high performances and extraordinary capabilities that are otherwise difficult or even impossible by natural materials.^[^ ^16^, ^17^, ^18^, ^19^ ^]^

**Figure 1:**
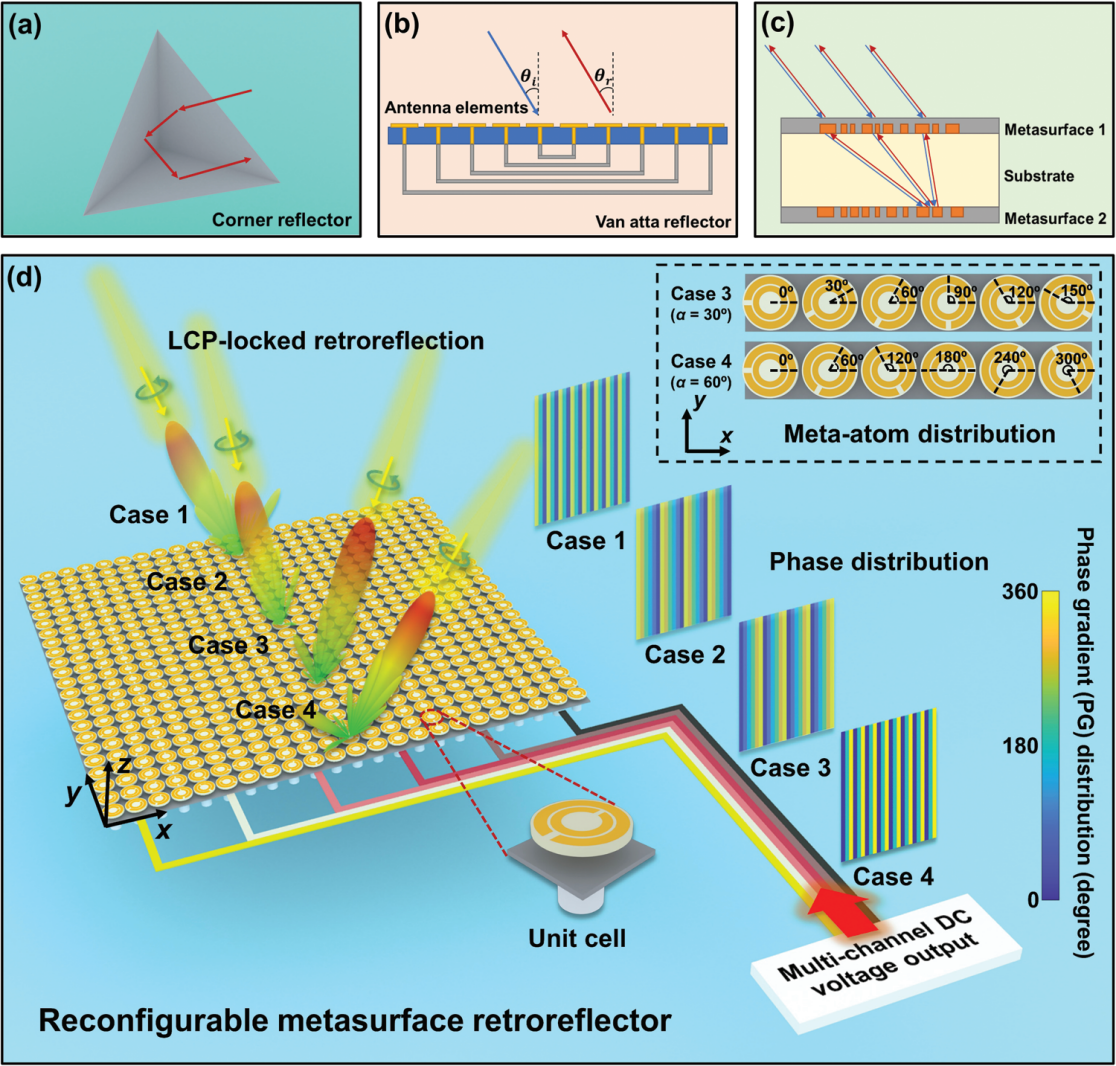
Schematic of current retroreflectors: a) bulky 3D metallic corner reflector, b) Van Atta type reflector, and c) cascaded metasurface pair for optical retroreflection. d) The proposed reconfigurable retroreflector based on adaptive phase gradient metasurface, where a continuous range of incidence angles of retroreflection can be realized by mechanically controlling the rotation angles of meta‐atoms. Such retroreflector can process either left‐handed or right‐handed circularly polarized (LCP or RCP) incidence (LCP cases are shown here, see Supporting Information for details of RCP cases), providing spin‐locked high‐efficiency retroreflection. Upper‐right panel shows the distribution of meta‐atoms for retroreflection case 3 (*α* = 30°) and case 4 (*α* = 60°).

Retroreflector, a device or surface that reflects incident EM wave back to the incoming direction over a continuous range of incidence angles, is one of the most important application of EM wave control as it has been widely used from microwave to light wave. In microwave region, a retroreflector typically acts as a device to improve its visibility by maximizing the backward scattering, while in optical region, it is highly desirable in laser tracking and optical systems. So far, devices like corner reflector (**Figure** **1**a), Luneburg lens, and cat's eyes reflector are implemented to realize retroreflection.^[^ ^20^, ^21^, ^22^, ^23^, ^24^ ^]^ However, these devices inevitably have limitations in miniaturization and integration with other components, because of their bulky and nonplanar structures. Planar version of retroreflector can be realized by Van Atta antenna array,^[^ ^25^, ^26^, ^27^, ^28^ ^]^ as shown in Figure 1b. It works as a passive self‐phasing antenna array, naturally reradiating the EM wave back toward the incident direction. Nevertheless, the complexity of bottom feed lines will be drastically improved as the increase of metasurface aperture, and such device only supports retroreflection for a certain single incident plane.

To tackle the above problems, the metasurface has been introduced to this field, but the current methods suffer from various limitations. The first metasurface optical retroreflector shown in Figure 1c is composed of two vertically stacked planner metasurfaces: the first layer performs a spatial Fourier transformation directing light with different incidence angles to different spots on the second metasurface; the second layer operates as a gradient metasurface and adds a spatially varying momentum equal to twice that of the incident light. Although the retroreflector has a planar structure, its overall thickness is still very large (about 590*λ*_0_).^[^ ^29^ ^]^ Then, a liquid‐metal‐injection method to change the structural parameters of C‐shaped meta‐atoms is proposed with a thin thickness of 0.2*λ*_0_.^[^ ^30^ ^]^ Such retroreflector represents a smart and practical method for individual and reversible control of meta‐atoms and demonstrates spin‐locked retroreflection at various angles. However, the stability is not reliable enough for measurement and transportation due to its fluidic nature, limiting its practical applications. The spin‐locked retroreflector composed of origami‐reconfigurable metagrating features high efficiency,^[^ ^31^ ^]^ however, it not only has a nonplanar profile but also is limited to a specific incidence plane with narrow range of incidence angles from 27.3° to 52.5°. Ideally, the reflection phase of individual meta‐atom should be controlled independently, continuously, and reversibly, which will greatly broaden the accessible range of incidence angles but clearly brings substantial challenges in the design due to the stringent requirements on arbitrarily tunable phase and high efficiency. For these reasons, it is still rare to simultaneously embrace the flat profile, high reflection efficiency, reconfigurability, and wide range of incidence angle in an ultrathin metasurface retroreflector.

Here, the objective is to seek for a new design approach to alleviate the abovementioned issues and simultaneously expand the retroreflection operations from a certain single incident plane to the omnidirectional half‐space. We propose a high‐efficiency, wide angle, and dynamically reconfigurable retroreflector composed of spin‐locked phase gradient metasurface with a thickness of only 0.07*λ*_0_ (*λ*_0_ is the operating wavelength at the center frequency of 4 GHz). The constituent meta‐atom is implemented based on the concept of Pancharatnam–Berry (PB) phase, whose reflection phase is determined by its structure orientation whereas the reflection handedness is kept the same as the incidence. Micromotor technique is used to rotate the meta‐atoms and then obtain the desired phase profiles on the metasurface. Micromotor technique has been proposed in the early mechanically phased reflectarray concept, which can eliminate the high cost of isolation network and phase shifters.^[^ ^32^ ^]^ Besides, the use of micro‐electromechanical systems (MEMS) micromotors can be applied for millimeter‐wave applications.^[^ ^33^ ^]^ With these merits, this technique of mechanically rotating the resonators to reconstruct the phase distributions can be used to achieve reconfigurable reflectarrays with beam scanning.^[^ ^34^, ^35^ ^]^ In this work, the structure orientation of each meta‐atom is individually controlled by a micromotor that is assembled behind the meta‐atom and electrically controlled by a field programmable gate array (FPGA) hardware system to realize dynamic reconfigurability, as shown in Figure 1d. Since each meta‐atom can be dynamically and continuously manipulated at will for circularly‐polarized wave, such metasurface retroreflector can provide desired spatially varying phases for arbitrary spin‐locked retroreflection. Moreover, we demonstrate that retroreflection in microwave frequency can be adaptively realized with high efficiency, not limited to a predefined incidence plane, but instead in a 3D wide range of incidence angles, by simply changing the electric signals applied to the micromotor array. Both experiments and simulations match well with each other. Therefore, we believe that the proposed thin thickness, high efficiency, and tunable metasurface retroreflector is expected to offer an untapped platform toward reconfigurable spin‐based retroreflection devices.

## 2 Principle and Element Design

To realize a phase gradient metasurface, an efficient way is to design meta‐atoms with different physical sizes and shapes, thus providing different resonant behaviors and required phase responses.^[^ ^1^, ^10^, ^36^, ^37^, ^38^, ^39^ ^]^ The geometric phase (also termed as PB phase) metasurface, however, can achieve full phase coverage by rotating anisotropic meta‐atoms with identical geometric parameters.^[^ ^35^, ^40^, ^41^, ^42^, ^43^ ^]^ The PB phase metasurface can offer great flexibility in the wavefront control just by using an array of identical inclusions with different geometric orientations, thus reducing the design complexity. Based on the concept of PB phase, here we design a metasurface retroreflector composed of C‐shaped resonators with same geometric parameters.

**Figure 2:**
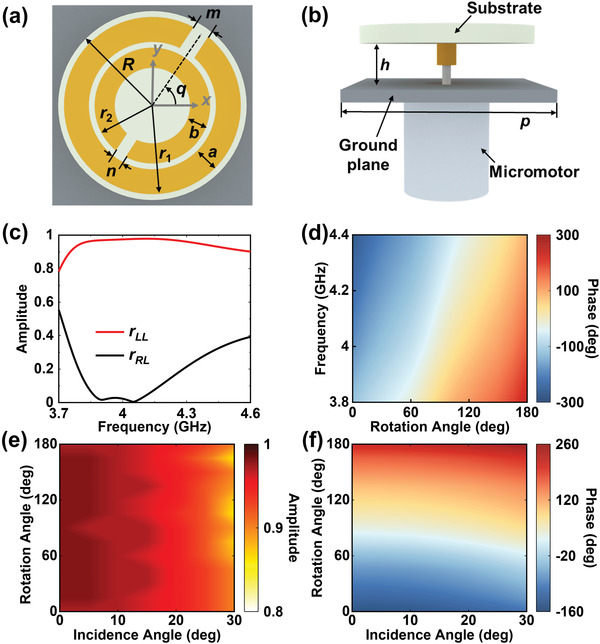
Schematic of the meta‐atom and its electromagnetic properties. a) The top view and b) side view of double C‐shaped meta‐atom loaded with micromotor. Simulated reflection c) amplitude and d) phase responses of the proposed meta‐atom under normal LCP incidence at different frequencies. Simulated copolarized reflection e) amplitude and f) phase for LCP incidence as functions of incidence angle and rotation angle at 4 GHz.

We start with the analysis of meta‐atom. When the double C‐shaped meta‐atom is rotated around its geometric center by an anticlockwise angle *q* with respect to *x*‐axis in *xoy*‐plane (**Figure** **2**a) and under the illumination of right‐handed and left‐handed circularly polarized (RCP and LCP) wave, denoted by $E_{i}^{R}$ and $E_{i}^{L}$ respectively, the reflection wave from the metasurface can be expressed as^[^ ^44^ ^]^

(1) $$E_{r}^{R}\;=r_{\mathrm{LR}}\;E_{i}^{L}\;+\;r_{\mathrm{RR}}e^{-j2q}E_{i}^{R}$$

and

(2) $$E_{r}^{L}\;=r_{\mathrm{RL}}E_{i}^{R}\;+\;r_{\mathrm{LL}}e^{+j2q}E_{i}^{L}$$

where $E_{r}^{R}$ and $E_{r}^{L}$ represent the reflected RCP and LCP electric field components. The first (second) subscript indicates the polarization state of the reflection (incident) wave, for example, *r*_LR_ and *r*_RR_ are the cross‐polarized and copolarized reflection coefficients for RCP incidence. For an ideal PB phase element, the cross‐polarized component should be suppressed as zero (*r*_RL_ = 0, *r*_LR_ = 0). Clearly, the handedness of reflection is kept the same as the incidence. Meanwhile, an additional PB phase of ±2*q* is imparted on the reflected wave, where the sign “+” and “−” corresponds to LCP and RCP incidence. By continuously rotating up to 180°, the meta‐atoms are able to achieve a continuous phase range of full 360°. Therefore, spin‐locked arbitrary reflection can be realized using a reflective phase gradient metasurface based on PB phase.

Upon the retroreflection, the relationship between reflection angle (*θ*_R_) and that of incident wave (*θ*_I_) can be calculated by the generalized Snell's law^[^ ^1^ ^]^

(3) $$\sin\theta_{R}=\sin\theta_{I}+\frac{1}{k_{0}n_{I}}\frac{d\Phi }{dx}$$

where *k*_0_ is the wave vector in free space, *n*_I_ is the refractive index of the host medium in incident region, $\frac{d\Phi }{dx}=\frac{2\pi }{L}=\frac{2\alpha }{p}$ is the phase gradient along the *x*‐axis, *L* is the period length of the metasurface covering 2*π* phase range, *α* is the rotation angle difference between the adjacent meta‐atoms, and *p* is the period length of the meta‐atom. When retroreflection occurs, namely the incidence is deflected to the source, such adaptive phase gradient metasurface should be designed to meet the condition of

(4) $$\theta_{R}=-\theta_{I}=\sin^{-1}\left(\frac{1}{2k_{0}}\frac{2\alpha }{p}\right)$$

where *θ*_R_ is the retroreflection angle. Its absolute value is equal to incidence angle *θ*_I_, and the negative sign is attributed to the inversed direction of input and output wave.

Retroreflection over a continuous range of incidence angles will be achieved, once the phase gradient distribution can be continuously tuned by rotating each PB meta‐atoms. Therefore, we can see that the key step to realize arbitrary retroreflection is to achieve tunable PB phase meta‐atom capable of providing full phase coverage. To this aim, we have designed an electrically controlled reconfigurable metasurface whose constituent PB meta‐atom can be freely tunable with arbitrary orientation. The schematic of the proposed retroreflector is shown in Figure 1d, where a FPGA‐based hardware system is used as the external controller to individually address each micromotor and control the orientation of each meta‐atom mechanically. The general steps to achieve dynamically adaptive retroreflection are: first, the incoming direction of the incidence is detected by an array of direction‐finding antennas;^[^ ^45^, ^46^ ^]^ second, the incidence direction is sent to the computer to calculate the required phase distribution for retroreflection; third, such phase distribution is converted to specific voltage for controlling the orientation of each meta‐atom. More details of the realization of dynamically adaptive retroreflection system can be found in Section S3 of the Supporting Information.

The designed meta‐atom is shown in Figure 2a,b. The top layer is a double‐C shaped copper pattern supported by a dielectric substrate. The bottom layer is an aluminum plate, acting as the ground plane to ensure a zero transmission of incidence. The micromotor is assembled under the ground plane to control the rotation angle of each resonator, as well as avoid unwanted EM influence to the resonant structure. The top layer of the meta‐atom is designed with circular structures, suitable for rotation that avoids collision with adjacent meta‐atoms. An air spacer is designed between the top and the bottom layer to place metallic pedestal linking the dielectric substrate and bottom micromotors. The metallic pedestal is fixed to the micromotor through a via hole drilled on the bottom aluminum plate. When applying appropriate number of pulses, the micromotor can convert the electrical pulse signal into the required step angle. For example, the inset figure in the upper‐right corner of Figure 1d shows the rotation angle distribution of meta‐atoms with *α* being 30°and 60° for case 3 and case 4, respectively. Based on the PB phase and Equation (4), spin‐locked retroreflection for continuous incidence angles can be realized by adjusting the phase gradient of the metasurface via tuning the rotation angle difference *α* between adjacent meta‐atoms by virtue of micromotors.

To explore the performance of the metasurface element, reflection behavior for circularly polarized incident wave is analyzed using the commercial software of CST Microwave Studio. Double C‐shaped resonator is chosen as the basic build blocking due to its dual‐resonance property that can broaden the bandwidth and reduce the electric size of meta‐atom effectively. The optimized geometrical parameters of the meta‐atom are: the radius of substrate is *R* = 8 mm, the radiuses of the outer and inner split rings are *r*_1_ = 7.5 mm and *r*_2_ = 4.9 mm respectively, the widths of the outer and inner split rings are *a* = 2.1 mm and *b* = 1.7 mm respectively, and other parameters are *m* = 1.5 mm, *n* = 1 mm, and *p* = 17 mm. The metallic pattern is made of copper (*σ* = 5.96 × 10^7^ S m^−1^) with a thickness of 0.018 mm. The dielectric of substrate is selected as Rogers RO4360G2 (*ɛ*_r_ = 6.15, tan*δ* = 0.0038) with a thickness of 1.524 mm, and the height of the air spacer between substrate layer and ground layer is set as *h* = 3.7 mm. Periodic boundary conditions are applied to *x* and *y* directions, and open boundary is applied to *z* direction. Figure 2c shows the simulation results of cross‐ and copolarized reflection coefficients under normal incidence of LCP wave. It showcases that the amplitude of copolarized reflection coefficient is close to 1 whereas that of cross‐polarized reflection coefficient is nearly zero within the frequency range from 3.75 to 4.6 GHz (relative bandwidth of 20.4%), approaching to the condition of forming an ideal PB element that ensures *r*_LL_ = 1 and *r*_RL_ = 0. To realize arbitrary retroreflection, 2*π* phase shift is needed to fully control the wavefront of the EM wave. We simulate the phase responses under LCP incidence with different meta‐atom rotation angles at several frequencies, as shown in Figure 2d. When the meta‐atom is anticlockwise rotated by an angle from 0° to 180°, the reflection phase is shifted twice the rotation angle and finally achieves full phase coverage. Moreover, such relationship can be observed within the frequency of interest, indicating good bandwidth performance. Meanwhile, the reflection amplitude always keeps close to 1, and immune from the change of rotation angle. Good angular stability is a key point for realizing high‐efficiency retroreflection especially for large‐angle incidence. Hence, we also investigate the angular performance of the meta‐atom at 4 GHz, as shown in Figure 2e,f. When the incidence angle of EM wave increases from 0° to 30° and the rotation angle changes from 0° to 180°, simulated copolarized reflection amplitude shows a high reflection with most cases higher than 0.9, showing a high‐efficiency operation. For phase responses, 2*π* phase shift coverage can be achieved for a wide incidence range. Although the reflection phase gradually changes as the incidence angle increases, the meta‐atom can still achieve the full 2*π* phase coverage, which means that the characteristic of PB phase can be well kept at oblique incidence. Therefore, retroreflection works for oblique incidence case as well, just by searching the design chart to slightly tune spatial distributions of the rotation angle.

The above results demonstrate that the proposed meta‐atom features high‐efficiency, wide‐angle, and flexible tuning of reflection phase for LCP illumination. Since the PB phase based element can work for different spins but with an inverse phase response, the meta‐atom also has high performance for RCP case. When illuminated by RCP incidence, the meta‐atom shows similar characteristics in terms of efficiency, bandwidth, angular performance, etc., as shown in Figure S1 of the Supporting Information. On the whole, we aim at engineering the retroreflection for LCP incidence as the design examples unless otherwise stated.

## 3 Retroreflection at Various Incidence Angles

**Figure 3:**
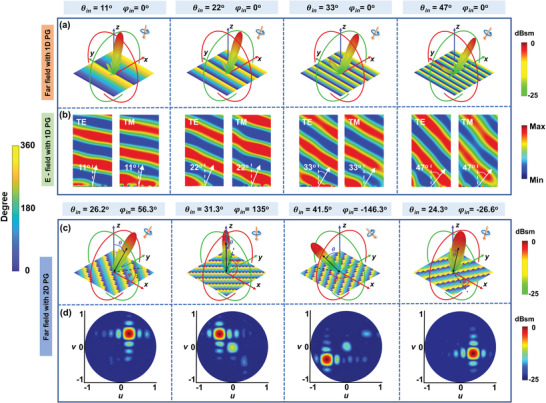
Simulated results of spin‐locked retroreflection. a) Far‐field LCP scattering patterns of spin‐locked metasurface retroreflector at different incidence angles for LCP incidence, and b) the corresponding cross‐polarized electric field distribution for TE (left panel) and TM (right panel) incidence. Simulated c) far‐field LCP scattering patterns of spin‐locked omnidirectional retroreflection from different directions for LCP incidence using the metasurface loaded with 2D phase gradient (2D PG), and d) corresponding 2D retroreflection scattering patterns in UV‐coordinate system.

To realize retroreflection while locking the spin characteristic of the incidence, here we use a metasurface consisting of 529 meta‐atoms, with each meta‐atom addressed by an electrically controlled micromotor. Based on Equation (4), the metasurface can be adaptive to different incidence angles by tuning the difference of rotation angle *α* between the adjacent meta‐atoms, which then leads to a change of the spatial phase profile on the metasurface aperture. Benefit from the excellent performance of meta‐atom at the operating frequency, spin‐locked retroreflection can be kept at various incidence angles. As a proof of concept, angular adaptive retroreflection in a predefined incident plane (*xoz*‐plane) at 4 GHz (*λ*_0_ = 75 mm) over a continuous range of incidence angles from 0° to 47° is demonstrated, as shown in **Figure** **3**. Figure 3a shows the simulated far‐field results of retroreflection behaviors for LCP wave at the incidence angles (the incident plane is *xoz*‐plane) of 11°, 22°, 33°, and 47°, corresponding to rotation angle difference *α* of 15°, 30°, 45°, and 60°. Obviously, the highly directive output beams are anti‐parallel to the incident direction, reflecting most of the incident energy back to the incoming direction. The 2D far‐filed scattering calibrated to the specular reflection of a same‐sized metallic plate is provided in Figure S2 (Supporting Information) to show the high‐efficiency of the retroflection. The maximum efficiency is about 92% when the incidence angle is 11°, whereas as the incidence angle increases, the retroreflection efficiency is gradually reduced. We also investigate the near‐filed distributions for straightforward observation, as shown in Figure 3b. Here, we define the transverse electric (TE)/transverse magnetic (TM) mode as the electric field/magnetic electric field perpendicular to the incidence plane. The left panels show the reflected TM component under the illumination of TE incidence, whereas the right panels show the TE component for TM incidence. In all cases, the incidence wave is mostly converted to its cross‐component, indicating that the metasurface retroreflector operates as a half‐wave plate, which also verify the property of spin‐locked retroreflection.^[^ ^30^ ^]^ Details of other components of electric field distribution can be found in Figure S3 of the Supporting Information. Although four incidence angles are demonstrated, we note that the rotation angle of the meta‐atom can be almost continuously rotated to realize arbitrary phase distribution, and as a result, the retroreflection within the angle range of 0° to 47° can be fully achieved. Retroreflection for RCP incidence can also be realized using this reconfigurable metasurface, just by reversing the rotating direction of the meta‐atom and the simulated results are illustrated in Figure S4 of the Supporting Information. We also analyze retroreflections at different frequencies from 3.8 to 4.4 GHz to see its bandwidth performances and the results are shown in Figure S5 of the Supporting Information. Clearly, accurate retroreflections can be achieved at four representative frequencies, indicating good bandwidth performance.

In real‐world scenarios, the incident EM wave may not come from a predefined incident plane but an arbitrary direction instead. Thus, it is significant to realize omnidirectional retroreflection for incidence with a wide angle range, which will undoubtedly improve the impact of the metasurface retroreflector. However, most of the current metasurface retroreflectors are limited to one certain polarization or one specific incidence plane,^[^ ^27^, ^28^, ^31^ ^]^ hindering their further uses in real‐world applications. This problem can be solved with the proposed metasurface retroreflector by applying 2D phase gradient to the metasurface. When the metasurface containing phase gradient along both *x*‐axis and *y*‐axis, the incidence plane to obtain retroreflection is no longer limited to *xoz*‐plane, but can be an arbitrary incident plane. In this case, the retroreflection angle can be written as

(5) $$\theta_{R}=\theta_{I}=\sin^{-1}\left(\frac{\lambda }{2\pi p}\sqrt{\alpha^{2}+\beta^{2}}\right)$$

where *α* and *β* represent the rotation angle difference between adjacent meta‐atoms along *x*‐ and *y*‐axes, respectively. Details of the derivation is provided in the Supporting Information. Since each meta‐atom and its reflection phase can be flexibly and individually controlled by the micromotor, it is easy to form 2D phase gradient interface by the proposed metasurface that is further used as a high‐efficiency retroreflector adaptive to EM wave with different incidence angles and incident planes.

As a proof of principle and to further verify the feasibility of the proposed metasurface, we introduce the 2D phase gradient to provide more degrees of freedom and perform full‐wave simulations to demonstrate the omnidirectional retroreflection at 4 GHz. The incidence direction is set as clockwise changed with respect to *z*‐axis, which successively comes from the first, second, third, and fourth quadrants with azimuth angle (denoted by *θ*) and elevation angle (denoted by *φ*) of (*θ*_1_,*φ*_1_) = (26.2°, 56.3°), (*θ*_2_,*φ*_2_) = (31.3°, 135°), (*θ*_3_,*φ*_3_) = (41.5°, −146.3°), and (*θ*_4_,*φ*_4_) = (24.3°, −26.6°). To achieve retroreflection, the meta‐atoms are rotated to form the required spatial phase distribution and the rotation angle difference between two adjacent meta‐atoms is sequentially set as *α*_*x*1_ = 20°, *α*_*x*2_ = −30°, *α*_*x*3_ = −45°, and *α*_*x*4_ = 30° along *x*‐axis, and *β*_*y*1_ = 30°, *β*_*y*2_ = 30°, *β*_*y*3_ = −30°, and *β*_*y*4_ = −15° for *y*‐axis. The far‐field LCP scattering patterns of metasurface retroreflector are shown in Figure 3c. The azimuth angle and elevation angle of reflected wave keep the same as that of the incidence, verifying the retroreflection effect by the proposed metasurface. At the same time, the high directive beams with low side lobes demonstrate the high operation efficiency. To give a clear view, we also plot in Figure 3d the 2D scattering patterns of metasurface by projecting the intensity distribution in 3D space vertically onto the 2D *uv*‐plane with the projection factors *u* = sin(*θ*)cos(*φ*), and *v* = sin(*θ*)sin(*φ*). Unambiguously, single dominating scattering beam successively appears in four quadrants of backward half‐space. The cross‐components of the reflected waves are shown in Figure S6 (Supporting Information), whose scattering energy is at least 5 dB lower than the copolarized components that demonstrate the spin‐locked characteristic. Therefore, such reconfigurable spin‐locked metasurface can be applied to achieve omnidirectional retroreflection by flexibly imparting 2D phase gradient.

## 4 Experimental Verification

**Figure 4:**
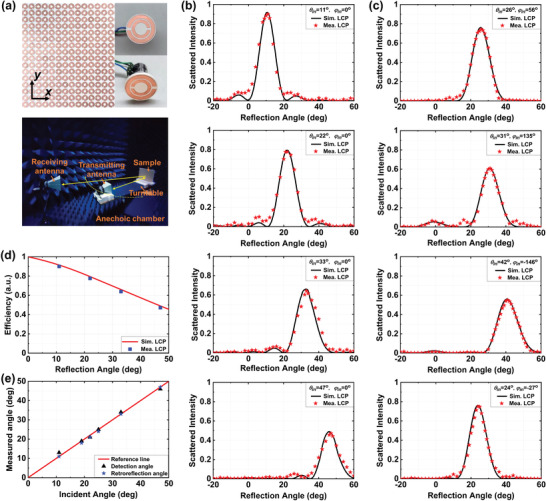
Experimental demonstration. a) Photograph of the Fabricated sample (upper) and test scenario (bottom). The inset of the upper picture shows a single meta‐atom whose ground plane is removed for a clear view. In the bottom picture the test sample, turntable, transmitting, and receiving antennas are placed in a standard microwave chamber. The blue pyramids are broadband microwave absorbing materials. b) The measured and simulated copolarized results of 2D scattering patterns at 4 GHz for various incidence angles in *xoz*‐plane (*φ*_in_ = 0°), with *θ*_in_ = 11°, 22°, 33°, and 47° shown in the upper panel to the bottom panel, respectively. c) Simulated and experimental results of the spin‐locked omnidirectional retroreflection at 4 GHz, with incidence angles of *φ*_1_ = 56°, *θ*_1_ = 26°, *φ*_2_ = 135°, *θ*_2_ = 31°, *φ*_3_ = −146°, *θ*_3_ = 42°, and *φ*_4_ = −27°, *θ*_4_ = 24° shown in the upper panel to the bottom panel, respectively. d) The efficiency of retroreflection at various angles for LCP incidence. e) The measured results of detection angles output from the direction‐finding antenna system and the corresponding retroreflection angles from the metasurface sample. Red line shows the ideal case where the measured angle equal to incidence angle.

The fabricated spin‐locked metasurface for adaptive retroreflection is shown in the upper panel of **Figure** **4**a. It is composed of 23 × 23 = 529 meta‐atoms with total size of 40 cm × 40 cm. The photograph of the individual meta‐atom loaded with micromotor is shown in the inset of Figure 4a, where the metallic ground plane is removed for a clear view of the metastructure and the micromotor. The micromotor used here in the metasurface is a two‐phase four‐wire stepper motor, which can be trigged by the DC voltage with a fast response time, and each micromotor is connected to a micromotor driver. Then, all of the micromotor drivers are linked to the I/O ports of the FPGA (AX301) based hardware controller. The maximum responding time of meta‐atom to rotate in the range of 0° to 180° is 2 s. More details of the whole response time of the dynamically angular‐adaptive retroreflection can found in Section S3 of the Supporting Information. Since each I/O port of the FPGA is individually and dynamically programmed, the metasurface can be reprogrammed to enable high‐efficiency retroreflection at various incidence angles.

The spin‐locked retroreflection is experimentally verified in a microwave chamber with microwave absorbing materials placed on the walls to get rid of unwanted microwave from the surrounding environment. The metasurface is placed at the center of an arc track with radius of 1.5 m, as shown in the bottom panel of Figure 4a. A pair of wideband double‐ridged horn antennas working as the emitter and receiver are connected to the two ports of a vector network analyzer (Agilent N5244). Both of the two horn antennas can provide RCP and LCP wave by a switching connector. One of them is mounted on the arc track, which serves as the emitting source to provide oblique incidence. The other horn antenna works as the receiver, standing behind the transmitting antenna to measure the co‐ and cross‐polarized scattered fields at various angles. When the transmitting antenna together with metasurface sample move along the arc track, the scattered fields reflected by the metasurface can be recorded by the receiver. Besides, it should be noted that in the measurement setup, the two antennas are placed on the two sides of the surface normal of sample with an angle of 5° in the vertical plane to avoid blockage of the antenna, and more details can be found in Section S5 of the Supporting Information.

We first demonstrate the metasurface retroreflector with 1D phase gradient for a certain incident plane. Figure 4b shows the experimental results of far‐field copolarized scattered intensity distribution for retroreflection at different angles of 11°, 22°, 33°, and 47° under the illumination of LCP wave in *xoz*‐plane. The reflection intensity and the total efficiency is normalized to the specular reflection of a same‐sized metallic plate under same incidence angle. The scattering patterns can exactly describe the efficiency of retroreflection, the spin‐locked characteristic, and the accurate reflection angles. To better show the performance of dynamic angular‐adaptive retroreflection, another two experimental examples of retroreflection around 22° are shown in the Figure S7 of the Supporting Information, where the incidence angles are 19° and 25° and the corresponding rotation angle difference *α* are two irregular values of 27° and 34°, verifying that the phase gradient distributions can dynamically follow the incidence angle. Considering the tolerance of fabrication and measurement, the experimental results are consistent with the simulated results in all of the four cases, proving the design principle and its feasibility. The measured efficiency of retroreflection at various angles for LCP incidence is shown in Figure 4d, which agree well with the simulated results (solid line). Clearly, retroreflection efficiency is relatively high when incidence angle is smaller than 20°, and gradually reduces as the increase of incidence angle. This is because the impedance mismatch brings a significant reflection to undesired direction or energy absorption at extreme oblique angles.^[^ ^47^, ^48^, ^49^ ^]^ Details of scattered intensity at various retroreflection angles under LCP and RCP incidence can be found in Figure S2 of the Supporting Information. Then, to prove the performance of omnidirectional retroreflection, the metasurface sample is reconfigured with 2D phase gradient by the external controller. As shown in Figure 4c, we experimentally verify the metasurface for retroreflection under different LCP incidence of *φ*_1_ = 56°, *θ*_1_ = 26°, *φ*_2_ = 135°, *θ*_2_ = 31°, *φ*_3_ = −146°, *θ*_3_ = 42°, and *φ*_4_ = −27°, *θ*_4_ = 24°, corresponding to the four designed examples of Figure 3c. The photograph of the second sample is shown in Figure S10 of the Supporting Information. Notably, the scattering pattern is illustrated in the incident plane, namely the cut plane of *φ* = 45°. It should be noted that the 3D far‐field scattering patterns cannot be directly measured in the current microwave chamber. Therefore, when measuring the results of omnidirectional retroreflection, the retroreflector sample should be rotated with an appropriate angle in its own plane so as to make the retroreflection plane parallel to the horizontal plane. As shown, the experimental result is in good agreement with the simulated result, verifying its capability of omnidirectional retroreflection. These results solidly verify the reconfigurable spin‐locked retroreflection for circularly polarized waves. The above results are measured based on the assembled test platform shown in the bottom picture of Figure 4a, which is used to measure the whole far‐field scattering pattern of the metasurface sample. Another test platform to directly measure the dynamically angular‐adaptive retroreflection system is shown in Figure S9 of the Supporting Information. A commercial direction finder (Rohde & Schwarz DDF007) is used to obtain the wave arrival information. We first measure the incidence angle and then input this angle into the predesigned program to automatically calculate the required phase gradient on the metasurface, and finally rotate the meta‐atoms using micromotors. Details of the working procedure is shown in Figure S8 (Supporting Information). The incidence angles, the detection angles output from the direction‐finding antenna, and the measured retroreflection angles are compared in Figure 4e. The red reference line represents the ideal results where the detection angles and retroreflection angles are almost equal to the incidence angles. It showcases that the maximum deviations of detection angles output from the direction‐finding antenna and the measured retroreflection angles are 2° and 1°, respectively. Therefore, the abovementioned results solidly demonstrate the accuracy performance of the dynamically angular‐adaptive retroreflection system, and the slight fluctuations is mainly caused by the fabrication, assembly, and measurement tolerance. Despite the advantages of flexible control, high efficiency and low profile, the mechanically driven element may also have some disadvantages concerning reliability and mass production. Several methods can be employed to improve its reliability. For example, scheduled recalibration of the stepping motors can improve the reliability caused by the accumulated error, and the implementation of algorithms of closed‐loop control can achieve a significant reduction of the motion delays.^[^ ^33^ ^]^ In addition, the well‐developed MEMS techniques may be integrated with metasurfaces to realize tunable metasurface and actively manipulation of EM wave, which may promote the mass production.

## 5 Conclusion

In conclusion, we propose an adaptive metasurface retroreflector for circularly polarized wave using spin‐locked phase gradient metasurface composed of PB phase meta‐atoms with a subwavelength thickness of 0.07*λ*_0_. Retroreflection for various continuous incidence angles can be realized by rotating the meta‐atoms that are controlled by the electrically driven micromotors. In particular, 2D phase gradients are introduced to achieve more degrees of freedom in controlling the reflection wave and largely extend the operation range of incidence angles. The simulated results have been verified by the microwave experiments, and good agreements are observed between them. Compared with the reported retroreflectors with bulky structures and limited freedom of retroreflection in a predefined plane, the proposed retroreflector possesses advantages of low profile, easy configuration, high efficiency, and large retroreflection angle in an omnidirectional half‐space. The design principle can also be readily extended to other frequency bands, such as millimeter waves, terahertz waves, by possibly using MEMS techniques, offering an untapped platform toward reconfigurable processing of spin waves.

## Conflict of Interest

The authors declare no conflict of interest.

## Supporting information

Supporting Information

Supplemental Movie 1

## Data Availability

The data that support the findings of this study are available from the corresponding author upon reasonable request.
