# Supplementary material for: Angular‐Adaptive Reconfigurable Spin‐Locked Metasurface Retroreflector
Source: Adv Sci (Weinh). 2021 Sep 5;8(21):2100885. doi: 10.1002/advs.202100885 (PMC8564442; doi:10.1002/advs.202100885)
Supplement: Supplementary file 1 — Supporting Information [file ADVS-8-2100885-s002.pdf]

© 2021 Wiley-VCH GmbH

ADVANCED SCIENCE Open Access

Supporting Information

for *Adv. Sci.,* DOI: 10.1002/advs.202100885

Title: Angular-Adaptive Reconfigurable Spin-Locked Metasurface Retroreflector

Weixu Yang, Ke Chen, Yilin Zheng, Wenbo Zhao, Qi Hu, Kai Qu, Tian Jiang, Junming Zhao, and Yijun Feng**

Dr. W. Yang, Dr. Y. Zheng, Dr. W. Zhao, Dr. Q. Hu, Dr. K. Qu, Prof. K. Chen, Prof. T. Jiang, Prof. J. Zhao, Prof. Y. Feng
Department of Electronic Engineering, School of Electronic Science and Engineering, Nanjing University, Nanjing, 210093, China
E-mail: ke.chen@nju.edu, yjfeng@nju.edu.cn

1. Simulated results of meta-atom and retroreflection

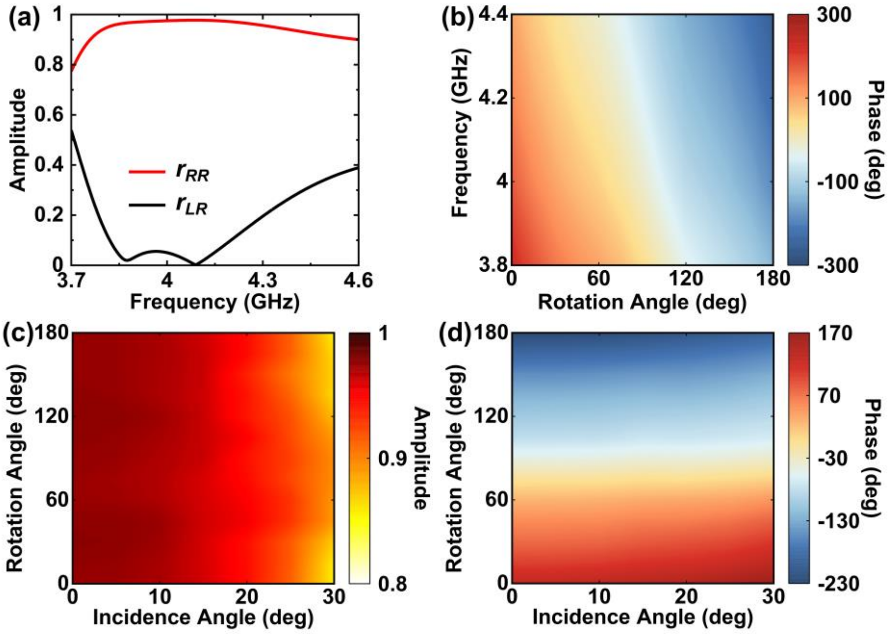

Figure S1. Simulated reflection c) amplitude and d) phase responses of the proposed meta-atom under normal RCP incidence at different frequencies. Simulated colormaps of co-polarized reflection e) amplitude and f) phase for RCP incidence as functions of incidence angle and rotation angle at 4 GHz.

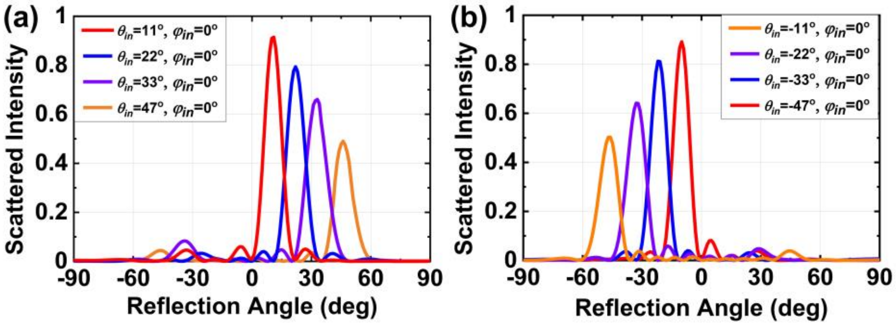

Figure S2. Simulated two-dimensional (2D) retroreflection patterns under c) LCP incidence and d) RCP incidence with various incident angles.

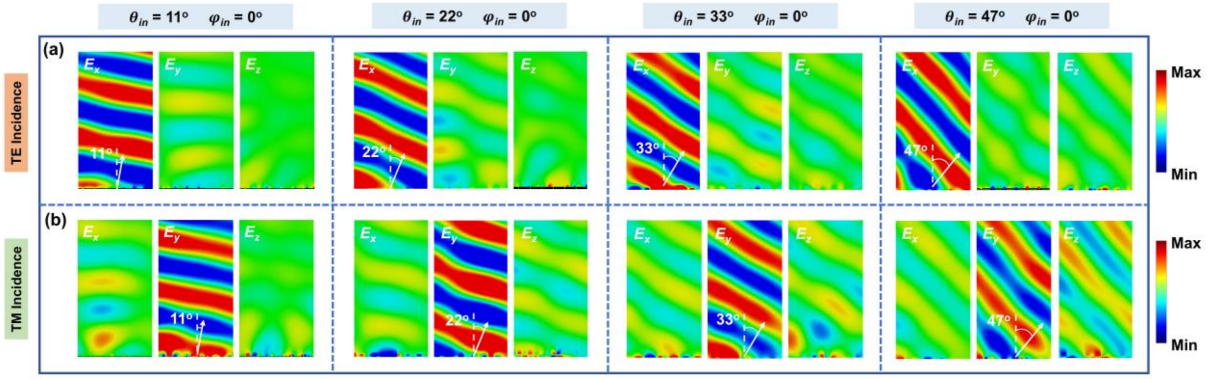

Figure S3. Simulated scattered electric field distribution at four different incidence angles for a) TE and b) TM incidence corresponding to Figure 3b.

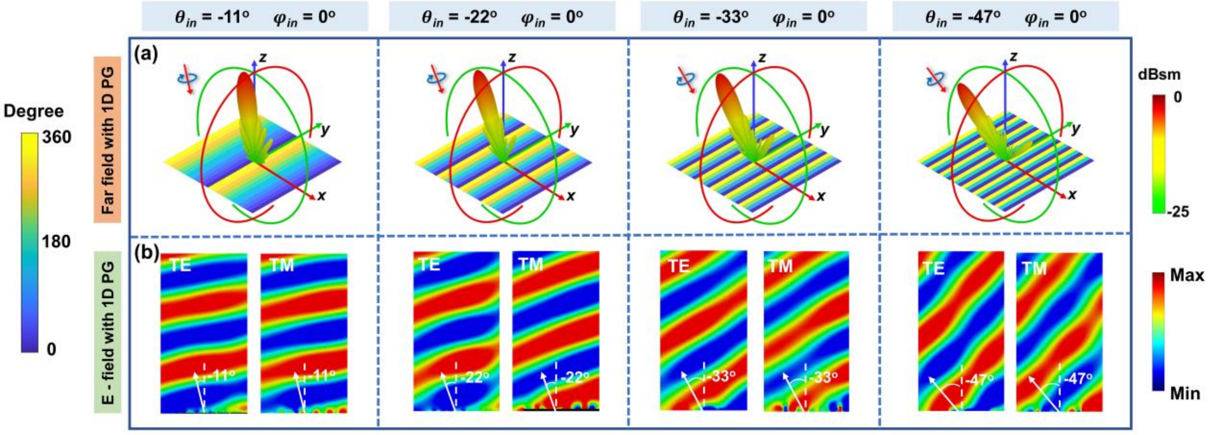

Figure S4. Simulated a) far-field RCP scattering patterns of spin-locked metasurface retroreflector at different incidence angles for RCP incidence, and b) the corresponding cross-polarized electric field distribution for TE (left panel) and TM (right panel) incidence.

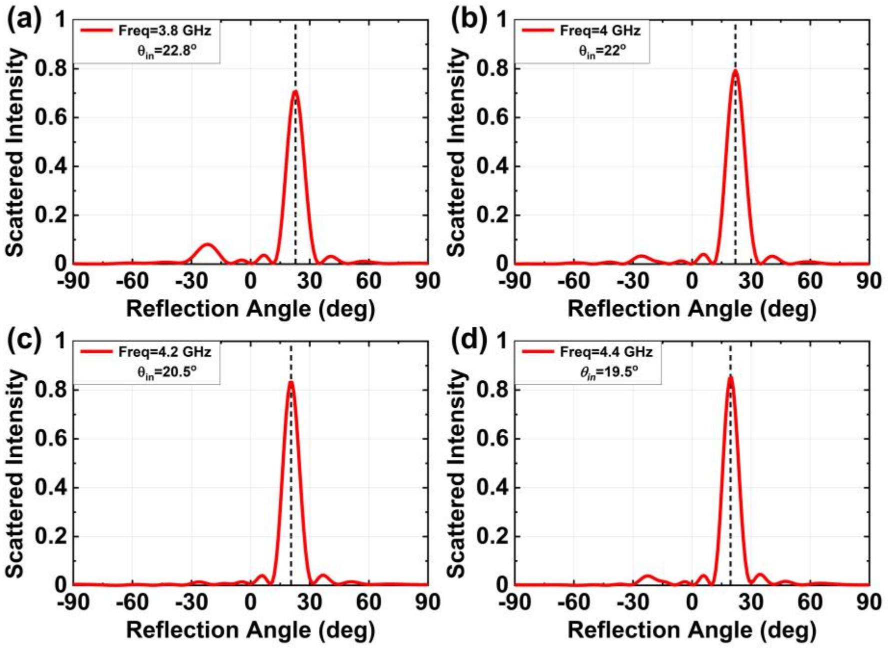

Figure S5. Simulated two-dimensional retroreflection patterns under LCP incidence at four different frequencies: a) 3.8 GHz, b) 4 GHz, c) 4.2 GHz and d) 4.4 GHz. The metasurface is with angle difference α of 30°. The black dotted lines represent the incidence angle.

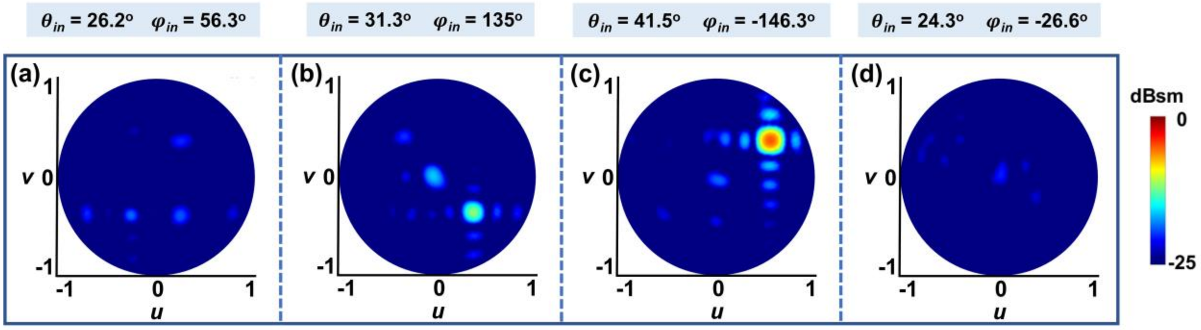

Figure S6. Simulated 2D scattering patterns of cross-polarized components for different spin-locked omnidirectional retroreflections under LCP incidence in Figure 3d. The incidence angles are a) (θ_1, φ_1) = (26.2°, 56.3°), b) (θ_2, φ_2) = (31.3°, 135°), c) (θ_3, φ_3) = (41.5°, -146.3°), and d) (θ_3, φ_3) = (24.3°, -26.6°), respectively.

2. Supplementary measured results of retroreflection

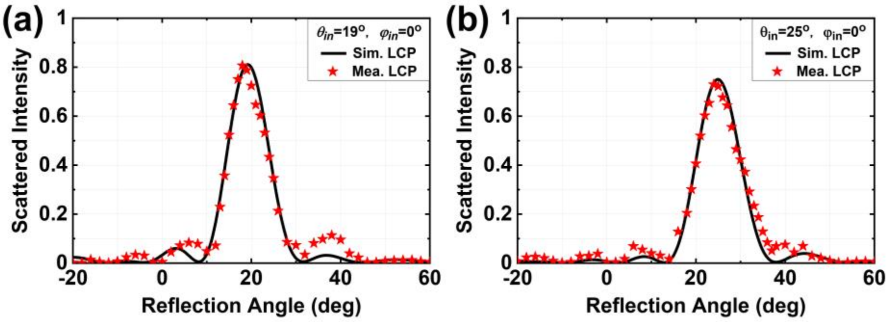

Figure S7. Simulated and experimental results of far-field co-polarized scattered intensity at 4 GHz for two examples of retroreflection around 22°: retroreflection for incidence angle in *xoz*-plane ($\varphi_{in} = 0°$) with a) θ_{in} =19° and b) θ_{in} =25°, corresponding to the rotation angle difference α being two irregular values of 27° and 34°, respectively.

3. Realization of dynamically angular-adaptive retroreflection system

To realize dynamic change of the metasurface retroreflector when the incidence angle is changed, a direction-finding (DF) antenna is employed to find the arrival direction of the incidence. The whole process of the dynamically angular-adaptive retroreflection system can be divided to three parts as described by the flow chart shown in Figure S8: the detection of incidence angle, the control system, and the dynamic response of retroreflection. In the detection part, we use a commercial direction finder ROHDE&SCHWARZ (R&S®) DDF007 to detect and determine the incidence angle of the transmitting antenna. Here, the transmitting antenna is placed away from the sample to mimic a plane wave incidence, as shown in Figure S9. The DF antenna R&S® ADD207 consists of two multi-element arrays mounted one above the other, and each array contains eight elements with operating frequency band from 690 MHz to 6 GHz. High-precision correlative interferometer DF method is used to guarantee the DF accuracy with a typical value of 1°. The working panel of R&S® DDF007 is shown in the inset of Figure S9, which can automatically output the measured information to the computer through network cables. In the second part of control system, we input manually the detected incidence angle to pre-designed program to calculate the required phase gradient distribution on the metasurface and generate the corresponding control signals by the FPGA (AX301) based hardware controller. The hardware controller can simultaneously output hundreds signals of bias voltage. Then, the control signals are transformed to the micromotor driver to control the rotation angle of each micromotor. Finally, the phase pattern of the reconfigurable metasurface can be reconstructed to achieve dynamic response of retroreflection to distinct incidence angles.

For the overall response time of the dynamically angular-adaptive retroreflection process, it is mostly determined by the response time of the stepper micromotor, and the times of the other two parts are negligible. For the first detection part, the incidence information can be detected by the direction finder R&S® DDF007 with about 10 ms and instantaneously transmitted to the computer. Although several seconds are required to manually input the incidence information to the pre-designed program in the current scheme, this process can be further improved when the two steps are linked automatically. Finally, the micromotor has a maximum overall response time of 2s, and thanks to the parallelization in control circuit board design, all meta-atoms are simultaneously rotated. Hence, the maximum control time of the whole system is about 2s if the detection and calculation program are connected.

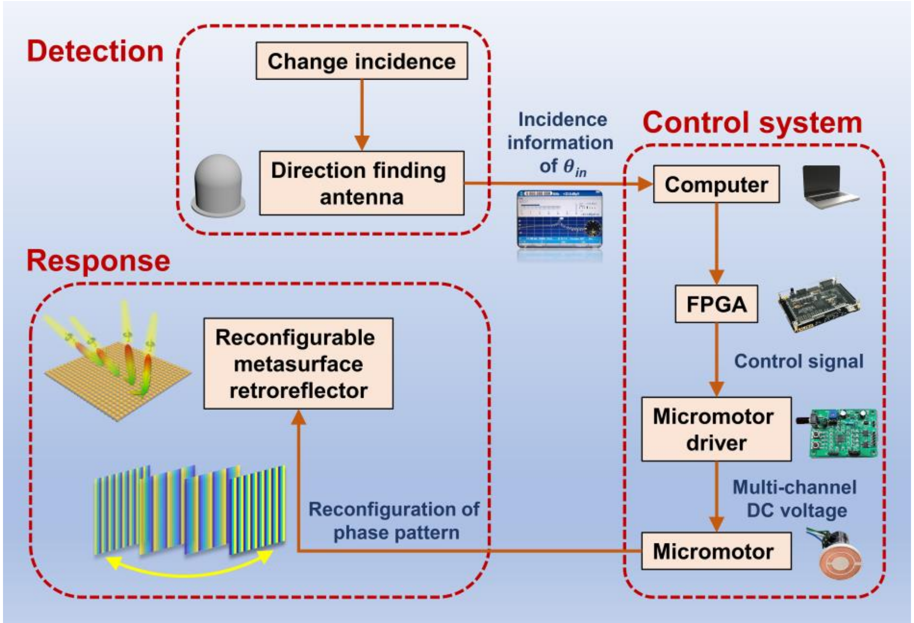

Figure S8. Flow chart of the realization of dynamically angular-adaptive retroreflection system

Based on the abovementioned discussion, the dynamically angular-adaptive retroreflection platform containing a commercial DF antenna is assembled, as shown in Figure S9. Here, the DF antenna is placed on the rotational axis of the turntable, and then the sample retroreflector is fixed on the above of the DF antenna to rotate synchronously. The transmitting and receiving antennas are placed with far-enough distance from the sample. The rotation of the turntable can mimic the change of the incidence angle. Therefore, different incidence angles can be automatically detected by the DF antenna system, as shown by the working panel of the device in the inset of Figure S9. Then, the dynamic retroreflection responses are performed by the reconfigurable retroreflector based on the detected information of incidence. The measured results of detection angles and retroreflection angles are depicted in the Figure 4e, where good agreements are found between them.

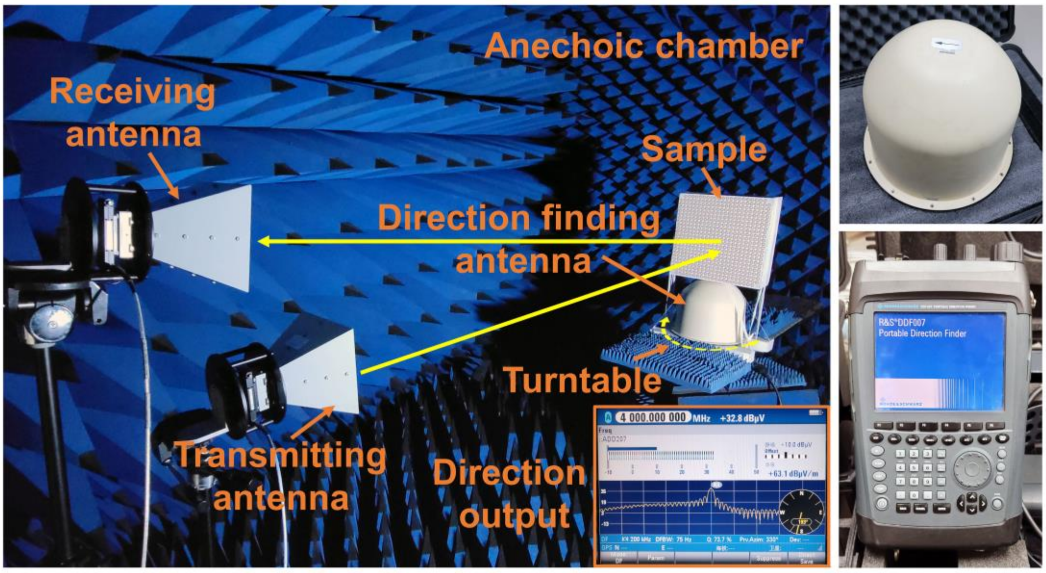

Figure S9. Photograph of the dynamically angular-adaptive retroreflection platform in a standard microwave anechoic chamber, where the test sample and DF antenna are placed on the axis of a turntable. The enlarged views of DF antenna (upper-right panel) and the portable direction finder R&S® DDF007 (bottom-right panel).

4. Derivation for retroreflection with two-dimensional (2D) phase gradient

According to generalized Snell's law, when the metasurface is designed with phase gradient along both *x*-axis and *y*-axis, the anomalous reflection wave will no longer be confined to the incidence plane. When such phase gradient metasurface is used to realize retroreflection, suppose the rotation angle difference between adjacent meta-atoms along x-axis is α, and that along y-axis is β, the 2D phase gradient can be derived as

$$\frac{\mathrm{d}\Phi}{\mathrm{d}r} = \sqrt{\left(\frac{2\alpha}{p}\right)^2 + \left(\frac{2\beta}{p}\right)^2}, \tag{S1}$$

where $\frac{\mathrm{d}\Phi}{\mathrm{d}r}$ is the 2D phase gradient, as shown in Figure S10. Obviously, they obey the addition theory of vector. Therefore, azimuth angle of retroreflection (or the projection of the incidence on the *xoy*-plane) is

$$\varphi = \tan^{-1}\left(\frac{\beta}{\alpha}\right). \tag{S2}$$

Based on equation (4), when retroreflection occurs, the pitch angle is

$$\theta = \theta_R = \theta_I = \sin^{-1}\left(\frac{\mathrm{d}\Phi}{2\mathrm{d}r \cdot n_I k_0}\right) = \sin^{-1}\left(\frac{\lambda}{2\pi p}\sqrt{\alpha^2+\beta^2}\right). \tag{S3}$$

So ideally, when the metasurface is designed with 2D phase gradient, retroreflection can cover the omnidirectional half-space. The incidence in Cartesian coordinate system can be derived as

$$\begin{cases} x = -\sin\theta\cos\varphi \\ y = -\sin\theta\sin\varphi \\ z = -\cos\theta \end{cases}. \tag{S4}$$

Then, for a given incidence, the needed phase gradient distribution for retroreflection can be calculated and transformed to the metasurface with meta-atoms rotated to pre-designed orientation. The rotation angle differences along two orthogonal axes for incidence with azimuth angle φ and pitch angle θ can be derived as

$$\begin{cases} \alpha = \sqrt{\dfrac{\left(\frac{2\pi p}{\lambda}\sin\theta\right)^2}{1+\tan^2\varphi}} \\ \beta = \sqrt{\dfrac{\tan^2\varphi\cdot\left(\frac{2\pi p}{\lambda}\sin\theta\right)^2}{1+\tan^2\varphi}} \end{cases}. \tag{S5}$$

Finally, the rotation states distribution of each meta-atom determined by α and β are dispatched to micromotors controlled electrically by the FPGA based hardware system, forming metasurface reflector with desired 2D gradient distribution.

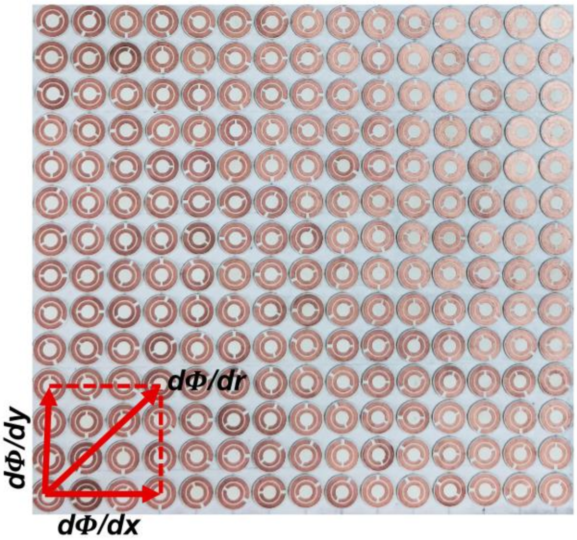

Figure S10. The fabricated sample of reconfigurable phase gradient metasurface loaded with two-dimensional phase gradient.

5. Details of the measurement setup

When measuring the RCS patterns of the retroreflector, the blockage of transmitting antenna will affect the measured performance of retroreflection if the two antennas are in the same horizontal plane, because the receiving and transmitting antennas are in the same direction. We did find this problem during the initial testing process and this problem will

eventually reduce the measured efficiency of retroreflection and increase the sidelobe level. To solve the problem, an improved measurement setup was used, as shown in Figure S11, which is the side view of the platform shown in the bottom panel of Figure 4a of the main text. The transmitting antenna and the assembled sample are fixed on the turn table to rotate in the horizontal plane together, while the sample itself can rotate along the vertical axis alone for mimicking different incidence angle. The transmitting and receiving antennas are placed on the two sides of the surface normal of the sample with an angle of 5° in the vertical plane, namely, an additional pitch angle along the vertical direction between the two antennas and the metasurface is used in the measurement. Because there is no phase gradient along the vertical direction on the metasurface, the oblique incidence will be reflected to specular direction in the vertical plane, which can be detected by the receiving antenna without any blockage in whatever directions. For 2D (omnidirectional) retroreflection cases, the sample should be rotate to enable the effective phase gradient parallel to the horizontal plane, which ensures that there is no phase gradient along the vertical direction.

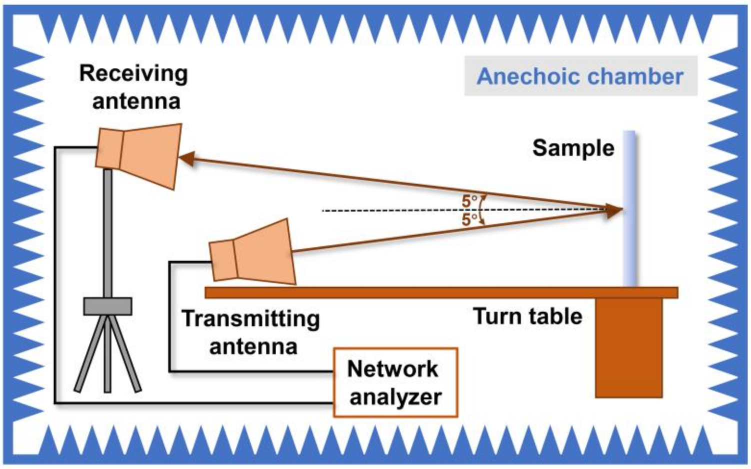

Figure S11. The schematic of the sideview of measurement setup shown in the bottom panel of Figure 4a.
